# Flowering Phenology Shifts in Response to Functional Traits, Growth Form, and Phylogeny of Woody Species in a Desert Area

**DOI:** 10.3389/fpls.2020.00536

**Published:** 2020-05-06

**Authors:** Yan Wang, Xiao-Dong Yang, Arshad Ali, Guang-Hui Lv, Yan-Xin Long, Ya-Yun Wang, Yong-Gang Ma, Chang-Chun Xu

**Affiliations:** ^1^Institute of Resources and Environment Science, Xinjiang University, Ürümqi, China; ^2^Department of Geography and Spatial Information Technology, Ningbo University, Ningbo, China; ^3^Key Laboratory of Oasis Ecology, Ürümqi, China; ^4^Department of Forest Resources Management, College of Forestry, Nanjing Forestry University, Nanjing, China; ^5^Co-Innovation Center for Sustainable Forestry in Southern China, Nanjing Forestry University, Nanjing, China

**Keywords:** fruit type, flower color, maximum plant height, phylogenetic signal, pollination mode, trees, shrubs

## Abstract

Climatic factors are considered the major driving forces for variation of flowering phenology among species. Yet, whether flowering phenology of woody species varies with functional traits, growth form, and phylogeny in arid regions is unknown. In the present study, we evaluated the relationships of three characteristics of flowering phenology (i.e., first flowering date, end of flowering date, and flowering duration) against functional traits, growth form, and phylogeny across 59 woody plant species across 3 years in Ürümqi city of the Xinjiang Autonomous Region, in Northwest China. The results showed that, plant functional traits and growth form had significant influences on the variability of flowering phenology among species. The contributions of fruit type (34.7–43.5%) and flower color (30.1–30.7%) to the variability of flowering phenology were larger than those of pollination mode (4.6–14.4%), life form (8.4–14%) and maximum plant height (9.7–13.1%). Trees had the significant correlations in terms of flowering duration against first flowering date and end of flowering date, while shrubs showed the opposite pattern. The values of phylogenetic signal (Blomberg’s *K*) of the three characteristics of flowering phenology ranged from 0.36 to 0.43, which were significantly lower than the expectation of the Brownian motion model. Our results suggested that functional traits, growth form and phylogeny all affected variability of flowering phenology among species. Our results provide a new perspective for correctly evaluating the relationship between global climate change and plant reproduction.

## Introduction

Flowering phenology, as the starting point of plant reproductive growth and an important phase of general phenology, is the core attribute of plants that allows them to cope with environmental changes and progeny reproduction (Cortés-Flores et al., 2017; König et al., 2017). Flowering phenology can reflect the influences of climate change on individual plant fitness and biodiversity through biological activities, such as pollination, seed diffusion, seed germination, and seedling settlement (Sun and Frelich, 2011; Davies et al., 2013). Previous studies have considered climate factors, such as temperature and sunshine period, as the main causes for variation of flowering phenology among species (Boyle and Bronstein, 2012; Davies et al., 2013). However, our understanding regarding the confounding influences of plant functional traits, growth forms, and phylogeny on flowering phenology remains unclear (Hu et al., 2015; Cortés-Flores et al., 2017).

Functional traits refer to a series of core adaptive strategies that are closely related to plant colonization, survival, growth, and mortality (Jia et al., 2011). Previous studies have shown that plant functional traits affected the variation of flowering phenology among species (Petersen et al., 2010; Sun and Frelich, 2011; König et al., 2017). This may be because the combination of flowering phenology and functional traits is the intrinsic strategy of plants to reduce the environmental limitation on reproductive success (Jia et al., 2011). Plants have evolved a multi-dimensional strategy, such as the variations on flowing phenology, fruit type, and flower color, to improve reproductive success (Kumar, 2016). However, there is an ongoing debate regarding which type of functional traits combined with flowering phenology improves reproductive success (Jia et al., 2011; Cortés-Flores et al., 2017). In other words, it is not clear which types of functional traits has an obvious relation with flowering phenology. For example, in tropical and sub-tropical forests, flowering phenology has obvious correlations with maximum plant height and pollination mode, while it is not significantly related to other traits (Sun and Frelich, 2011; Hu et al., 2015). In humid temperate forests, flower color and pollination mode make an obvious manipulation on the variability of flowering phenology among species, whereas fruit type and maximum plant height do not (Dowding, 1987; Lönnberg, 2004; Jia et al., 2011). Changes in environmental constraints among different habitats might be one of the major explanations for the above ongoing debate (Freschet et al., 2013; Fontana et al., 2017). In tropical and sub-tropical forests, taller plants and diverse pollination modes are conducive to reducing the constants of high stand density and multi-layered vertical structure on reproduction success (Selaya et al., 2007; Bashir et al., 2013). Variabilities in flower color and pollination mode are beneficial to decrease the influence of climate disasters (such as cold snaps) on reproduction success via shortening the pollination time in humid temperate forests (Jia et al., 2011; Bawa, 2016). Drought, low temperature, high temperature difference, and frequent climate disasters are the major environmental constraints in the arid desert ecosystem, which differ from tropical, sub-tropical, and humid temperate forests. Thus, the type of functional traits connecting with the variation on flowering phenology in arid desert regions would differ from those in the other types of forest. However, most previous research studied the relation of functional traits with flowering phenology in humid habitats (Du and Qi, 2010; Hu et al., 2015). Whether and which type of functional traits connect with flowering phenology in arid desert regions have not been studied.

The phylogenetic constraint hypothesis suggests that variability of flowering phenology among species might be determined by phylogeny (Vidiella et al., 1999; Jennings, 2001; Du et al., 2015). Phylogenetic conservatism and phylogenetic relationships determine that phylogenetically closely-related species have the same adaptive strategies to cope with environmental constraints (Davies et al., 2013; Du et al., 2015). However, the constraint of phylogeny on flowering phenology have been mainly conducted on larger scales (i.e., country, climate zone, the northern hemisphere, and the global scales), and whether it affects variation of flowering phenology on the small scale, such as city and community scales, remains unclear (Davies et al., 2013; Du et al., 2015; Yang et al., 2018). Besides, according to the theory of phylogenetic niche conservatism, competitive exclusion plays a dominant role in the assembly of flowering phenology on the small scale, probably because of fierce resource competition (Kraft and Ackerly, 2010). As a result, this drives the divergence of phylogeny and the high dispersion of phylogenetically closely-related species on the small scale (Kraft and Ackerly, 2010). Flowering phylogeny might not be constrained by phenology on the small scale (Boyle and Bronstein, 2012), which deserves further study.

Variability of flowering phenology among species may be related with growth forms (Yang et al., 2018). Compared with shrubs, trees height give them advantages in terms of light interception and wind pollination for improving reproductive success (Boyle and Bronstein, 2012). Such environmental advantages might drive different strategies of flowering phenology between trees and shrubs. In addition, the temperature around the tree crown is lower than that around shrubs because of vertical temperature differences (Boyle and Bronstein, 2012; Yang et al., 2018). Long-term environmental selection would induce higher sensitivity of trees to temperature change than shrubs (Chávez-Pesqueira and Núñez-Farfán, 2016; Yang et al., 2018). In early spring, trees are sensitive to temperature increases, and then obtain more benefits from wind pollination (Du et al., 2015). However, there is little evidence to support the relationship between growth form and flowering phenology in arid desert regions.

In the present study, three characteristics of flowering phenology, i.e., first flowering date, end of flowering date, and flowering duration, of 59 woody plants were observed across three consecutive years to explore the relationships among flowering phenology, functional traits, growth form, and phylogeny in Ürümqi city in the Xinjiang Autonomous Region of China. The three main objectives of this study were: (1) To test whether and which type of functional traits connect with the variability of flowering phenology among species; (2) to test whether phylogeny affect the variability of flowering phenology among species; and (3) to test whether growth form affect the variability of flowering phenology among species.

## Materials and Methods

### Study Site

This study was conducted in Ürümqi city in the Xinjiang Autonomous Region, in northwest China. The site has a typical arid continental climate, with annual average precipitation of less than 294 mm, while the annual average evaporation is about 2500 mm. The precipitation distributions are uneven among the four seasons, with summer accounting for 50% of the annual total precipitation. The average maximum temperature (25.7°C) occurs in July and August, whereas the average minimum temperature occurs in January (−15.2°C). Plants begin to germinate in late March. Flowering duration lasts from mid-April to mid-late May. Extreme drought conditions mean that most plants in this site are drought-tolerant and cold-tolerant deciduous species.

### Collection of Flowering Phenological Data

The flowering phenology of 59 native woody plants (38 tree and 21 shrub species) in Ürümqi was recorded from early April to early June across three consecutive years (2016, 2017, and 2018) (Supplementary Table 1). Here 3-years data were treated as the experimental replication. We selected 59 native woody plants to eliminate the influence of the cultivated non-indigenous species on the phylogenetic constraint of flowering phenology. Before the observation of flowering phenology, three healthy individuals of each species were selected randomly and then labeled. All labeled plants were observed every 2 days to record three characteristics of flowering phenology. The date when more than 25% of buds in a single plant blossomed was recognized as the first flowering date. The date when less than 10% of buds in a single plant blossomed was determined as the end of flowering date. The time difference between the first flowering date and the end of flowering date was defined as the flowering duration (Zu et al., 2000).

### Collection of Plant Functional Traits and Growth Form

Previous studies indicated that flower color, fruit type and pollination mode, are the factors with the most connection with flowering phenology because they are the basic properties of flower and fruit, and play the most immediate roles in reproductive success (Petersen et al., 2010; Sun and Frelich, 2011; König et al., 2017). As a basic functional trait, maximum plant height affected the pollination efficiency and resource utilization, thus affecting flowering phenology (Sun and Frelich, 2011; Yang et al., 2018). Based on these understandings, maximum plant height, flower color, fruit type, and pollination mode were as selected our research objects to explore their relationships with flowering phenology. In this study, maximum plant height, flower color, and fruit type were collected by searching the *Flora of Xinjiang* eBook and *Flora of China* database^1^. Due to the variability of climate factors, such as precipitation and temperature, maximum plant height might be different between these above two databases. In this study, maximum plant height was preferentially obtained from *Flora of Xinjiang*. For some species, maximum plant height was collected from *Flora of China* while their values were not recorded in *Flora of Xinjiang.* Pollination mode was grouped into three sub-categories based on descriptions of basic flowering characteristics: insect-pollinated, wind-pollinated, and bird- pollinated modes. The species having large flowers, bright color, and obvious perianth patches adopted the insect-pollinated mode, while having those having fragrance-free, small flowers, and many stamens adopted the wind-pollinated mode (Rabinowitz et al., 1981). The bird-pollinated mode was not included in this study because their proportion accounted for less than 5% of the total number of species. According to the types of pericarp at maturity, the fruit types were divided into two sub-categories: fleshy (drupe, berry, and pome) and dry fruit. Dry fruits were further divided into dry dehiscent (capsule and pod) and dry indehiscent (samara and nut) fruit (Gordon, 1998). The main difference between fleshy and dry fruits was that fleshy fruit contain a fleshy pericarp at maturity whereas dry fruit contain a hard, papery, or dry pericarp at maturity. The main difference between dry dehiscent and dry indehiscent fruits was that the dry dehiscent fruit opened at maturity to discharge their seeds whereas dry indehiscent fruit did not open at maturity to discharge seeds. The cones and follicles of the fruit type were not considered because their species accounted for less than 5% of the total number of species. Flower colors were grouped into six sub-categories: white (pale white, yellow-white, and silver-white), red (pink, reddish, and fuchsia), brown (puce, taupe, tawny, and lavender brown), yellow (amber and pale yellow), green (white-green and yellow-green), and purple (mauve). Orange and first white and then yellow flowers were removed because their species also accounted for less than 5% of the total number of species. In this study, growth forms were divided into two sub-categories based on plant potential size: trees and shrubs. Among the categories of growth form (i.e., life history strategy of a plant, potential size, life-span, woodiness of a taxon), potential size (i.e., trees and shrubs) was selected as our studied object because it was the most important basic growth form in natural and semi-natural habitats (Liira et al., 2008). Growth form was also obtained by searching the *Flora of Xinjiang* eBook and *Flora of China* database^1^. Trees were defined as a plant having a permanently woody main stem or trunk, ordinarily growing to a considerable height, and usually developing branches at some distance from the ground. Shrubs were defined as a woody plant smaller than a tree, usually having multiple permanent stems branching from or near the ground.

### Data Analysis

For data analysis, the recorded date of flowering phenology was converted into Julian day (Hu et al., 2017). After that, based on all data points of 3 years, the following statistical methods were adopted to address three objectives of this study.

(1) Hierarchical partitioning analysis, generalized linear model (GLM), and the *post hoc* test were used to test the influences of functional traits and growth form on flowering phenology. More specifically, hierarchical partitioning analysis was first used to obtain the contributions of four functional traits (maximum plant height, flower color, fruit type, and pollination mode) and growth form (tree and shrub) to the variability of flowering phenology among species. The contribution is the proportion of each independent variable from the goodness-of-fit measures across all variable combinations in a hierarchy (Chevan and Sutherland, 1991). Then, GLM was used to test specific relationships of flowering phenology against sub-categories of each type of functional trait. In a level of a given type of functional trait, if one sub-category is not retained in GLM (NA), this indicates this sub-category has little influence on flowering phenology. Compared with the not-retained sub-category, the positive and negative estimates indicated that a sub-category has a later and earlier flowering phenology, respectively. The *post hoc* test was used to compare the differences in the influences of different sub-categories of a given type of functional trait on flowering phenology.

(2) Phylogenetic tree and one phylogenetic signal, i.e., Blomberg’s K, were used to test the constraint of phylogeny on the variation of flowering phenology among species. Species classification was matched to the Angiosperm Phylogeny Group III classification system [APG III (Angiosperm Phylogeny Group III), 2009]. The phylogenetic tree was constructed using the software Phylomatic (Version 3) (Webb et al., 2008). The values of Blomberg’s K were calculated using a Brownian motion model based on the phylogenetic tree. *K* = 1 was the expectation of the Brownian motion model indicating obvious phylogenetic conservatism, whereas *K* = 0 indicates no phylogenetic conservatism (Davies et al., 2013). However, when *0* < *K* < *1*, phylogenetic conservatism was determined by the significance of the difference between actual and random simulated phylogenetic signals. The significance of difference was tested using a simple randomization procedure, as implemented in Blomberg’s K calculations. In brief, an observed trait distribution on a phylogenetic tree was compared with the trait distribution randomly shuffled across the tips of that phylogeny. The null hypothesis in this analysis was that closely related species do not share similar patterns, representing *K* = 0 (Blomberg et al., 2003). In the present study, the number of repetitions was set as 999, where *P* < 0.05 indicated that phylogeny restricts the variability of flowering phenology among species. In this study, only 57 woody species were used to construct the phylogenetic tree (Supplementary Figure 1) and to calculate the values of Blomberg’s K because two gymnosperm species (*Juniperus rigida* and *Pinus sylvestris*) were not included in APG III tree. (3) Similar to the functional traits, GLM and the *post hoc* test were also used to test the relationship between flowering phenology and growth form. Additionally, Standardized Major Axis Estimation (SMA) was used to test the difference in the relationships in terms of three characteristics of flowering phenology between trees and shrubs (Warton et al., 2006). Tests for heterogeneity of regression slopes and calculation of common slopes where homogeneity of slopes was demonstrated followed Warton and Weber (2002).

All data analyses were conducted in R. 3.4.3. Hierarchical partitioning analysis was evaluated in *rdacca.hp* package. The values of Blomberg’s K and the *post hoc* test of GLM were evaluated in the *phytools* and *emmeans* packages, respectively. SMA was evaluated in *smatr* package.

## Results

### The Influences of Functional Traits on the Variability of Flowering Phenology Among Species

**FIGURE 1:**
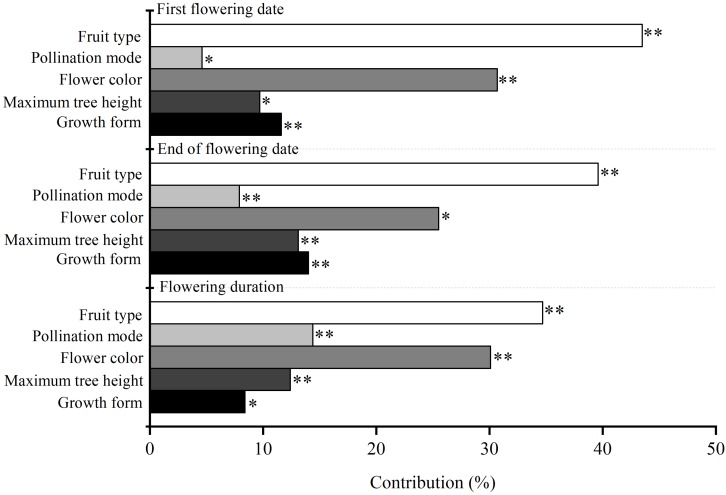
The independent contribution of plant functional traits (i.e., maximum plant height, flower color, fruit type, and pollination mode) and growth form (i.e., tree and shrub) to three characteristics of flowering phenology. ^∗∗^ and ^∗^ are the confidence coefficient of the functional traits to its corresponding flowering phenology in the hierarchical partitioning analysis. ^∗∗^*P* < 0.01; ^∗^*P* < 0.05.

Our results showed that the Julian days of first flowering date, end of flowering date and flowering duration of 59 woody species were 117 ± 2.16, 130 ± 1.93, and 13 ± 1.21, respectively (Supplementary Table 1). Maximum plant height, flower color, fruit type, and pollination mode all significantly affected the variability of flowering phenology among species (*P* < 0.05) (Figure 1). Fruit type made the highest contribution to variability of three characteristics of flowering phenology among species (34.7–43.5%), flower color made a moderate contribution (30.1–30.7%), and pollination mode made the smallest contribution (4.6–14.4%) (Figure 1).

*R*^2^ in GLM of first flowering date, end of flowering date and flowering duration were 0.62, 0.63, and 0.59, respectively. All *P*-value were < 0.001 (Table 1). At the level of flower color, yellow, red, green, and purple had significantly negative influences on first flowering date (*P* < 0.05), whereas brown had a non-significant influence (*P* > 0.05); however, white was not retained in GLM (NA). The *post hoc* test suggested that the influence of white on the first flowering date differed significantly from that of yellow (*P* < 0.05), while the differences were non-significant compared with red, purple, brown, and green (*P* > 0.05). At the level of fruit types, pod had a significantly positive influence on the first flowering date, whereas drupe, berry, capsule, nut, and pome had non-significant influences (*P* > 0.05); however, samara was not retained in GLM. The *post hoc* test suggested that the influences of samara on the first flowering date differed significantly with those of pod, berry, and capsule (*P* < 0.05), while the differences were non-significant compared with drupe, nut, and pome (*P* > 0.05) (Table 1). Additionally, fleshy fruit (drupe and pome) had negative influences on the first flowering date, while dry fruit (capsule, pod, and nut) had positive influences (Table 1).

**TABLE 1:** Difference in the influences of sub-categories of functional traits (i.e., maximum tree height, flower color, fruit type, and pollination mode) and growth form (i.e., tree and shrub) on the variation of three characteristics of flowering phenology among species.

Coefficients		First flowering date			End of flowering date			Flowering duration		
		Estimate	*P*	Result of the *post hoc* test	Estimate	*P*	Result of the *post hoc* test	Estimate	*P*	Result of the *post hoc* test
(Intercept)		4.92	<0.001***		4.99	<0.001***		2.40	<0.001***	
Growth form	Trees	–0.05	0.21	a	–0.05	0.19	a	–0.04	0.69	a
	Shrubs	NA	NA	a	NA	NA	a	NA	NA	a
Maximum tree height		–0.003	0.19		–0.003	0.17		–0.003	0.65	
Flower color	White	NA	NA	a	NA	NA	a	NA	NA	a
	Brown	–0.14	0.15	ab	–0.10	0.26	ab	0.14	0.58	a
	Red	–0.09	<0.05*	ab	–0.09	<0.05*	ab	–0.14	0.18	a
	Yellow	–0.22	<0.001***	b	–0.17	<0.001***	b	0.18	0.10	a
	Green	–0.10	<0.1*	ab	–0.11	<0.1*	ab	–0.17	0.30	a
	Purple	–0.12	<0.05*	ab	–0.11	<0.1*	ab	–0.02	0.88	a
Fruit type	Samara	NA	NA	a	NA	NA	ab	NA	NA	a
	Drupe	–0.12	0.12	a	–0.08	0.28	a	0.29	0.14	ab
	Pod	0.13	<0.1*	c	0.13	<0.1*	c	0.15	0.45	ab
	Nut	0.03	0.57	a	0.04	0.43	a	0.17	0.29	a
	Berry	0.06	0.50	bc	0.07	0.41	ab	0.14	0.54	ab
	Pome	–0.09	0.22	ab	–0.06	0.38	ab	0.21	0.29	ab
	Capsule	0.03	0.70	bc	0.08	0.25	bc	0.54	<0.01**	b
Pollination mode	Wind	–0.01	0.93	a	–0.04	0.59	a	–0.28	0.14	a
	Insect	NA	NA	a	NA	NA	a	NA	NA	a
Model statistics	*F* test (*P-*value)		835.36 (<0.001)			968.62 (<0.001)			83.96 (<0.001)	
	*R*^2^		0.62			0.63			0.59	

Specific relationships of flowering phenology against sub-categories of each type of functional trait were calculated using a generalized linear model (GLM). Differences in the influences of different sub-categories of a given type of functional trait on flowering phenology were compared using the *post hoc* test of GLM. NA indicates that the sub-category of a given type of functional trait and growth form is not retained in GLM, indicating this sub-category has little influence on flowering phenology. Lowercase letters are the result of the *post hoc* test of GLM. Different lower case letters between sub-categories at a given type of functional trait indicate a significant difference in the influence of those sub-categories on flowering phenology, whereas same lowercase indicates no significant difference (*P* < 0.05). ***P < 0.001; **P < 0.01; *P < 0.1.

At the level of flower color, yellow, green, red, and purple had significant negative influences on the end of flowering date (*P* < 0.05), whereas brown did not (*P* > 0.05), and white was not retained in GLM. The *post hoc* test suggested that the influence of white on the end of flowering date differed significantly from that of yellow (*P* < 0.05), while there were non-significant differences compared with purple, red, brown, and green (*P* > 0.05). At the level of fruit type, pod had a significant positive influence on the end of flowering date, whereas drupes, nut, berries, pome, and capsules did not (*P* > 0.05); however, samara was not retained in GLM. The influence of samara on the end of flowering date differed significantly from that of pod (*P* < 0.05), whereas it was not significantly different with that of drupe, nut, berry, pome, and capsule (*P* > 0.05) (Table 1). Additionally, fleshy fruit (drupe and pome) had negative influences on the end of flowering date, whereas dry fruits (capsule, pod, and nut) had positive influences (Table 1).

At the level of flower color, white was not retained in GLM, and the other sub-categories had no significant influences (*P* > 0.05). The *post hoc* test indicated that the influence of white on flowering duration was not significantly different from other sub-categories of flower color (*P* > 0.05) (Table 1). At the level of fruit type, samara was not retained in GLM, while capsule had a significant influence on flowering duration (*P* < 0.01), whereas drupe, pods, nut, berry, and pome had no significant influence (*P* > 0.05). The influence of samara on flowering duration was significantly different from that of capsule (*P* < 0.05), whereas it was not significantly different from that of the other sub-categories of fruit types (*P* > 0.05) (Table 1).

At the level of pollination mode, wind pollination had a non-significant influence on the three characteristics of flowering phenology (*P* > 0.05), whereas insect pollination was not retained in GLM. The influence of wind pollination on flowering duration differed non-significantly with insect pollination (*P* > 0.05) (Table 1).

### Phylogenetic Conservatism of Flowering Phenology

The values of Blomberg’s K of the three characteristics of flowering phenology ranged from 0.36 to 0.43, which were lower than the expectation of the Brownian motion model (Table 2). Our results showed a significant phylogenetic signal (all *P* < 0.05) in all three characteristics of flowering phenology (Table 2), suggesting moderate phylogenetic conservatism for the phenology of the 57 native woody plants tested.

**TABLE 2:** The values of Blomberg’s K of three characteristics of flowering phenology in 57 woody species.

Characteristics of flowering phenology	Blomberg’s K	*P*-values
First flowering date	0.39	<0.05
End of flowering date	0.43	<0.05
Flowering duration	0.36	<0.05

**P*-values show the significance of the difference between actual and random simulated phylogenetic signals. *P*-values < 0.05 indicate that phylogeny restricts the variability of flowering phenology among species.*

### The Influences of Growth Form on Variability of Flowering Phenology Among Species

Growth form had a significant influence on the variability of flowering phenology among species (*P* < 0.05). The contribution of growth form (8.4–14.0%) was lower than that of fruit type (34.7–43.5%) and flower color (30.1–30.7%) (Figure 1). Trees had a non-significant influence on the three characteristics of flowering phenology (*P* > 0.05), whereas shrub was not retained in GLM. The influence of trees on flowering duration was not significantly different from that of shrubs (*P* > 0.05) (Table 1). Additionally, trees had significant positive correlations among first flowering date, end of flowering date, and flowering duration (*P* < 0.01).

**FIGURE 2:**
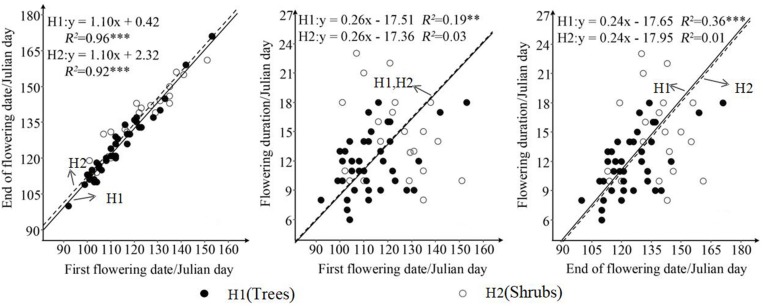
Differences in relationship in terms of three characteristics of flowering phenology between trees and shrubs. H1 and H2 represent trees and shrubs, respectively. ^∗∗∗^*P* < 0.001; ^∗∗^*P* < 0.01.

SMA results showed that first flowering date has a significant regression relationship with end of flowering date both for trees (*R*^2^ = 0.96; *P* < 0.001) and shrubs (*R*^2^ = 0.92; *P* < 0.001) (Figure 2). In the condition of common slope, the difference in the intercept between shrubs (2.32) and trees (0.42) was only 1.90. More importantly, this difference indicated that with the same date of first flowering, the end of flowering date of shrubs was later than that of trees. In other word, under the condition of the same date of first flowering, the flowering duration of trees was 1.90-days shorter than shrubs (Figure 2). In the condition of common slope, trees had the significant correlations in terms of flowering duration against first flowering date (*R*^2^ = 0.19, *P* < 0.01) and end of flowering date (*R*^2^ = 0.36, *P* < 0.001), while shrubs showed the opposite pattern (*P* > 0.05) (Figure 2). *R*^2^ of SMA for the relationships of flowering duration against the first flowering date and end of flowering date were 0.03 and 0.01, respectively (Figure 2).

## Discussion

### Fruit Type and Flower Color Made Larger Contributions to the Variability of Flowering Phenology Than the Other Functional Traits

Functional traits had a significant influence on the variability of flowering phenology among species; however, the contribution differed among their types (Figure 1). Fruit type made the highest contribution to the variability of flowering phenology among species (Figure 1). This might be because fruit type is the paramount and the most direct trait in determining plant reproductive success (Chen et al., 2017). During the process of plant growth, all changes in phenology are inclined to increase reproductive success (Rathcke and Elizabeth, 1985). After comparing the difference in the influences of sub-categories of fruit types on flowering phenology, our results showed that dehiscent fruit (pod and capsule) had a significant influence on flowering phenology, whereas indehiscent and fleshy fruit (samara, drupes, berries, nut, and pome) had little or no influence (Table 1). Additionally, the influence of dehiscent fruit on flowering phenology differed from that of indehiscent and fleshy fruit (Table 1). These results indicated that dehiscent fruit had larger influences on flowering phenology than indehiscent and fleshy fruit. This could be attributed to the differences in seed separation from fruit among sub-categories of fruit types (Lorts et al., 2008). In the extreme environment, dehiscent fruit expel their seeds immediately when ripe, while indehiscent fruit release seeds by decay *in situ*, or they are eaten by an animals and the seeds pass out intact in their droppings, resulting in advantages in germination for dehiscent fruit because of the prompt contact of seeds with soil (Lu et al., 2010). Pericarp decay and animal consumption are also the two main determining factors for seed separation of fleshy fruit; however, their contributions were higher than indehiscent fruit because of the fresh edible pericarp (Correia et al., 2016; Pérez-Méndez et al., 2018). Thus, fleshy fruit had an intermediate influence on flowering phenology compared with that of dehiscent and indehiscent fruit. Additionally, our results showed that fleshy fruit have a negative influence on flowering phenology, whereas dry fruit have a positive influence (Table 1), suggesting that fleshy fruit have an earlier flowering phenology than dry fruit. This was probably because of the difference in fruit size between fleshy and dry fruit. Compared with dry fruit, fleshy fruit bloom out and the flowers fade early to extend the fruit growth time to develop larger fruit (Hughes et al., 1994).

Flower color was another strong contributor to the variability of flowering phenology among species (Figure 1). This may be because flower color is the second most important influencing trait in terms of plant reproductive success (Toleno et al., 2010). Among sub-categories of flower colors, bright colors (yellow, red, purple, and green) had an obvious influence on the first flowering date and the end of flowering date, whereas inconspicuous colors (white and brown) had little or no influence (Table 1). In addition, the influence of the bright colors on the first flowering date and the end of flowering date differed obviously from that of inconspicuous colors (Table 1). These results suggested that the variation in first flowering date and the end of flowering date among species were more easily affected by bright colors. This could be determined by the difference in pollination mode between bright- and inconspicuous-colored flowers in spring (Bawa, 2016). Bright colored flowers attract insects more easily for pollination, while also benefiting from the wind; therefore, the production of the bright flower colors would increase reproductive success (Dowding, 1987). Unlike their effects of the first flowering date and the end of flowering date, bright colors did not influence flowering duration significantly compared with that of inconspicuous colors (Table 1). This may be because flowering duration is a derived variable from the first flowering date and the end of flowering date (equaled to the difference between the first flowering date and the end of flowering date). A slight change in the response of flower color to the end of flowering date or the first flowering date would attenuate the difference between bright and inconspicuous colors. This could also be reflected by the different influences of sub-categories of growth form and pollination mode on flowering duration. In this study, the influence of sub-categories of growth form and pollination mode on flowering duration was also not significantly different among the categories (Table 1). However, the influence of fruit type on flowering duration differed significantly among the sub-categories (dehiscent, indehiscent, and fleshy fruits) (Table 1). This could be because the contribution of fruit types was larger than that of flower color, growth form, and pollination mode (Chen et al., 2017). A slight change in the different sub-categories of fruit was not enough to attenuate the difference in their influence on flowering duration. Additionally, our results showed that all colored flowers had negative estimates in GLM (Table 1), indicating that colored flowers have an earlier flowering phenology than white flowers. According to the relationship between flower color and pollination mode, colored flowers are pollinated by both insects and the wind, whereas white flowers are mainly pollinated by the wind (Dowding, 1987). Shortages of insect activities caused by low temperature mean that wind is considered the main pollination mode in the North Temperate Zone in early spring (Whitehead, 1969; Bale et al., 2002; Hu et al., 2017). Thus, the early flowering phenology of colored flowers was conducive to making up the shortages in insect activities (Dowding, 1987; Hu et al., 2017).

Pollination mode also plays an important role in the variation of flowering phenology among species (Figure 1). Compared with insect pollination, wind pollination mode had a larger influence on the variabilities of the three characteristics of flowering phenology (Table 1). This might be determined by the limitations of geographical location and climatic environment (Dowding, 1987). Our study site was located in the North Temperate Zone, which has a low temperature and usually suffers from cold snaps in early spring (Johnson, 1993; Munguía-Rosas et al., 2011). Munguía-Rosas et al. (2011) reported that low temperature and cold snaps were not conducive to insect activities, thus delaying the flowering phenology of insect-pollinated species. In other words, wind-pollinated species can reproduced irrespective of weather conditions, while the reproduction of insect-pollinated species is limited by the weather (Chambó et al., 2017). In addition, our study site was also subjected to westerlies (a prevailing west wind) all year, resulting in an advantage for the reproductive success of wind-pollinated species compared with insect-pollinated species (Bale et al., 2002; Hu et al., 2017). This supported by the results of GLM, in which wind pollination had a negative estimate compared with insect pollination (Table 1), indicating that the flowering phenology of wind pollination was earlier than that of insect-pollination (Dowding, 1987). Such advantages would contribute to the early completion of pollination, and extend the growth time of seeds, resulting in high reproductive success (Jia et al., 2011). Interestingly, the contribution of pollination mode on flowering duration is much more than that on the first flowering date and the end of flowering date (Figure 1). This may be determined by the relationship between reproductive success and pollination mode (Barman et al., 2018). Compared with the first flowering date and the end of flowering date, the feedback of pollination mode on flowering duration has a greater influence on the reproductive success (Rabinowitz et al., 1981). To some extent, pollination mode determines the magnitude of flowering duration. For example, in the North Temperate Zone, the advantage of pollination efficiency enables wind-pollinated flowers to have a shorter flowering duration than insect-pollinated flowers (Jia et al., 2011).

### Phylogenetic Constraint on Variation of Flowering Phenology Among Species

The three characteristics of flowering phenology had significant phylogenetic signals across the 57 native woody plants, indicating that phylogeny constrained flowering phenology at the small scale (Table 2). However, our result was opposite to those of previous studies, which showed that phylogeny exerted little constraint on flowering phenology because of competition exclusion at the small scale (Kraft and Ackerly, 2010). More specifically, at small scales, in similar niches, interspecific competition plays a more important role in species composition of a community than habitat selection, thereby promoting the spatial separation of phylogenetically closely-related species (Kraft and Ackerly, 2010). In this case, the phylogenetic relationships among species at the small scale areas usually considered as divergence. Phylogeny has little constraint on the variability of flowering phenology among species (Kraft and Ackerly, 2010). However, during geological history, local species of the Xinjiang Uygur Autonomous Region were produced mostly after the Quaternary glacial period (Kochmer and Handel, 1986; Johnson, 1993). In addition, the uplift of the Qinghai-Tibet Plateau caused the evolutionary isolation of local species (Du et al., 2015). Unlike most places at the small scales, species had closer phylogenetic relationships in the Xinjiang Uygur Autonomous Region, which resulted in significant constraints of phylogeny on flowering phenology.

### Differences in Flowering Phenology Between Trees and Shrubs

Maximum plant height made less significant contributions to the variability of flowering phenology among species compared with fruit types and flower color (Figure 1), which suggested that plant height could influence flowering phenology indirectly via promoting the advantages of pollination and fruit type (Cortés-Flores et al., 2017). For example, taller plants are more likely to be easily pollinated by the wind, resulting in increased reproductive success (Jia et al., 2011). This was supported by our results that growth form made the smaller contribution to the variability of flowering phenology among species (Figure 1). Based on the maximum plant height, species were divided into two kinds of growth form: trees and shrubs. Trees had a larger influence on flowering phenology than shrubs, probably because trees had height advantages in obtaining light and photosynthesis to support their reproductive success (Boyle and Bronstein, 2012; Yang et al., 2018). Also, a negative estimate in GLM for trees suggested that trees had an earlier flowering phenology compared with shrubs (Table 1). Earlier flowering phenology would improve trees’ reproductive success because of the extended growth time of fruit (Bolmgren and Cowan, 2008; Jia et al., 2011). In addition, the contribution of maximum tree height to the variabilities of both the first flowering date and end of flowering date was much than that of growth form (Figure 1), whereas showed the opposite pattern for flowering duration (Figure 1). This may be caused by the data type. Maximum tree height and growth form all can be considered as the index of plant height. However, growth form was a qualitative binary variable (trees and shrubs), while the maximum tree height was a continuous variable. In the process of hierarchical partitioning analysis, contribution is the proportion of intra-group variance of each independent variable to total variance of all variable combinations (Chevan and Sutherland, 1991). In terms of mathematical logic, the transform of plant height into binary variables would increase its proportion in the total variance, thus resulting in a higher contribution of growth form to the first flowering date and end of flowering date compared with maximum tree height (Figure 1). But for flowering duration, since its magnitude reflected the time difference between the first flowering date and end of flowering date (Zu et al., 2000). The variation of the tradeoff between these two original traits across different species may reduce the proportion of intra-group variance of binary variable to total variance. In this case, the contribution of growth form on flowering duration was less than that of maximum tree height (Figure 1).

Differences in adaptive strategies of phenology between trees and shrubs could also be tested from the SMA results (Figure 2). Vertical temperature differences and long-term environmental selection mean that trees are more sensitivity to increased temperature than shrubs in spring (Boyle and Bronstein, 2012). Long-term adaptability caused trees to adopt an early flowering strategy (Johnson, 1993; Yang et al., 2018). In the North Temperate Zone, the advantages of wind-pollination would result in earlier flowering helping trees to compete pollination quickly. As a result, an earlier flowering strategy would drive trees to have an earlier end of flowering date and a shorter flowering duration (Figure 2) (Bolmgren and Cowan, 2008; Lessard-Therrien et al., 2014; Yang et al., 2018). Thus, trees had significant positive correlations with the first flowering date, the end of flowering date, and flowering duration (Figure 2). By contrast, reductions in the frequency and intensity of wind during the development season, the shift of the main pollinator from wind to insects, and the low efficiency of insect pollination would cause late flowering shrubs to take longer to complete pollination (Bolmgren and Cowan, 2008; Lessard-Therrien et al., 2014; Yang et al., 2018). However, nutritional consumption and the unpredictable risks (such as insect attack and cold snap), would increase the flowering duration correspondingly, resulting in a trade-off among flowering phenology, nutritional consumption, and unpredictable risks (Johnson, 1993; Peñuelas et al., 2004; Munguía-Rosas et al., 2011). Shrubs tend to have a later end of flowering date and a stable flowering duration (Fenner, 1998; Peñuelas et al., 2004). Therefore, shrubs had no significant correlations in terms of flowering duration compared with the first flowering date and the end of flowering date (Figure 2).

In this study, we found that the variability of flowering phenology among species was related to phylogeny and functional traits. In nature, plants often adopt a multidimensional adaptive strategy to reduce the environmental limitation on reproductive success (Kumar, 2016). For example, our study found that maximum tree height, flower color, fruit type, and pollination mode have the significant correlations with flowering phenology. This suggested that plants may have adopted a combination of flowering phenology, maximum tree height, flower color, fruit type, and pollination mode to increase reproductive success in an arid desert region. Among environmental factors, climate factors such as temperature and sunshine period were considered to be the most important determinants of reproductive success (Meng et al., 2017; Zhou et al., 2018). Climatic factors significantly affected the variation of flowering phenology among species (Chang-Yang et al., 2013; Qi et al., 2015). In this case, functional traits may be an intermediate variable in the influence of climate factors on flowering phenology. Or, the combination of functional traits and flowering phenology might act as an intermediate variable in the influence of climate factors on reproductive success. In addition, our study conducted on a city/small scale, resulting in the limitation on result expansion at the large scale. However, we did not assess the influence of climate factors on flowering phenology in this study. Therefore, in order to fully reveal the cause of the variation of flowering phenology among species, it is necessary to integrate reproductive success, climate, functional traits, phylogeny, and flowering phenology into a whole analysis, and explore the underlying ecological processes among them at large scale in future.

## Conclusion

The results of the present study indicated that plant functional traits and growth form contribute to the variation of flowering phenology among species in an arid desert region. Fruit type and flower color are the main functional traits that contribute to the variation of flowering phenology among species. Phylogenetic constraints also affect moderately variability of flowering phenology among species. The relationships of flowering duration with the first flowering date and the end of flowering date differ between trees and shrubs. Our results showed that functional traits, growth form, and phylogeny all affect the variability of flowering phenology among species. In the past few decades, global warming altered plant reproductive success, and then affecting plant biomass and diversity maintenance. The influence of global warming on plant reproduction was often overstated because it wasn’t considered the compensations of functional traits, growth form, and phylogeny to flowering phenology. Our results extended the basic theory of phenology, and provided a new perspective for correctly evaluating the relationship between global warming and plant reproduction.

## Data Availability Statement

The data used in this paper can be seen in the supporting materials Supplementary Table 1.

## Author Contributions

All authors designed the study. YW, Y-XL, Y-YW, and X-DY collected the data. YW, AA, G-HL, Y-XL, Y-YW, Y-GM, C-CX, and X-DY quantified recordings, ran statistical analyses, and drafted the manuscript. All authors read, revised, and approved the manuscript.

## Conflict of Interest

The authors declare that the research was conducted in the absence of any commercial or financial relationships that could be construed as a potential conflict of interest.

## Footnotes

1 http://iplant.cn/frps
