# Supplementary material for: Flowering Phenology Shifts in Response to Functional Traits, Growth Form, and Phylogeny of Woody Species in a Desert Area
Source: Front Plant Sci. 2020 May 6;11:536. doi: 10.3389/fpls.2020.00536 (PMC7219254; doi:10.3389/fpls.2020.00536)
Supplement: Supplementary file 1 [file Data_Sheet_1.doc]

Supplementary Material

# Supplementary Figure and Table

## Supplementary Figure


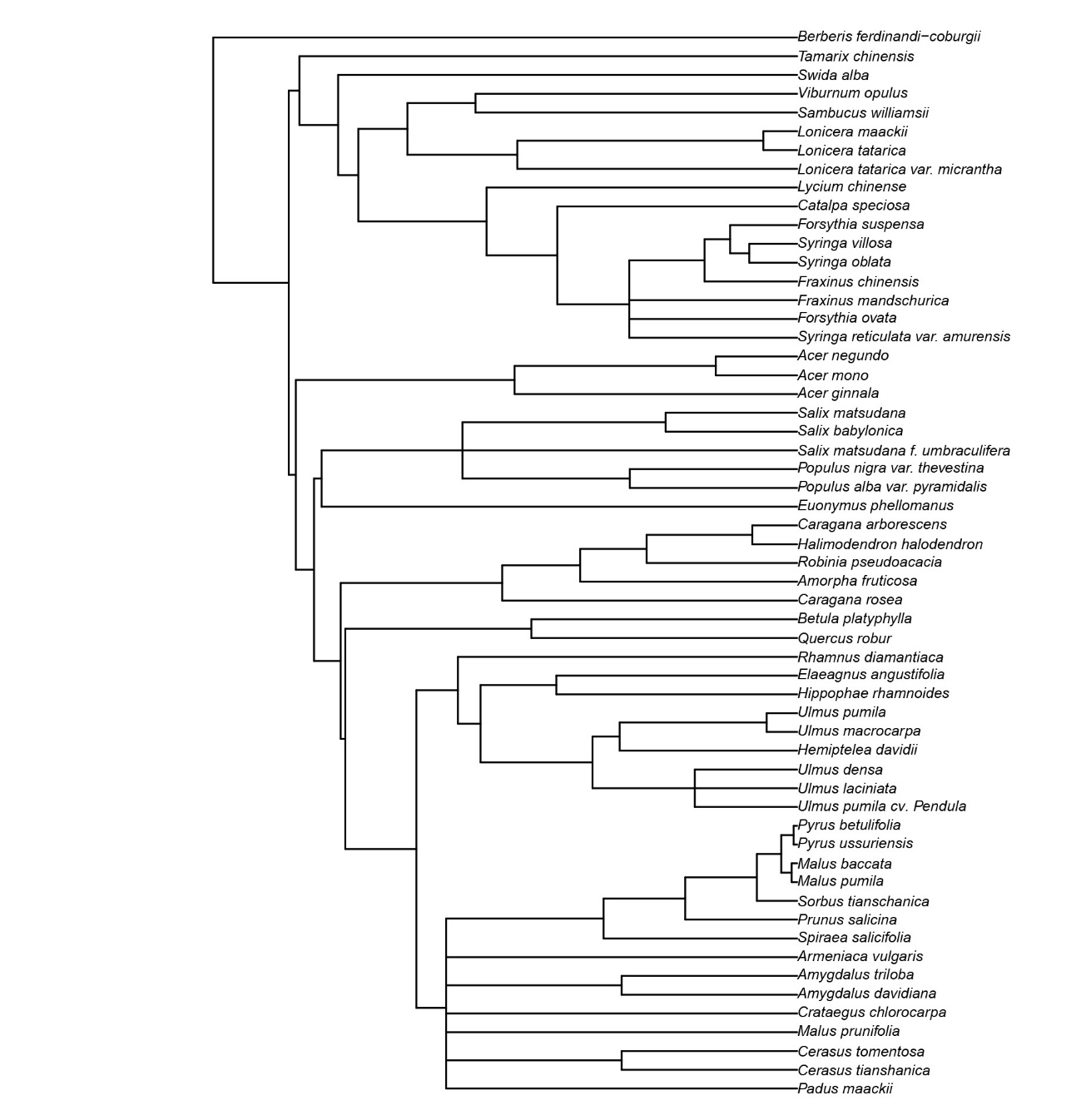


**Supplementary Figure 1.** The phylogenetic tree of 57 native woody species in Urumqi city, Xinjiang Autonomous Region.

## Supplementary Table

**Supplementary Table 1. Flowering phenology, growth form and functional traits of 59 woody plants in Urumqi city, Xinjiang Autonomous Region. Flowering phenology is the mean of three consecutive years. Julian days (*Mean*±*SD*).**

| Species name | Growth form | Functional traits | | | | Flowering phenology (Mean ± SD) | | |
| --- | --- | --- | --- | --- | --- | --- | --- | --- |
|  |  | Maximum plant height | Flower color | Fruit type | Pollination mode | First flowering date/d | End of flowering date/d | Flowering duration/d |
| *Malus pumila* | Tree | 15 | White | Poem | Insect | 111 ± 0.94 | 121 ± 0.82 | 10 ± 1.25 |
| *Malus baccata* | Tree | 14 | White | Poem | Insect | 112 ± 4.32 | 126 ± 1.25 | 14 ± 3.30 |
| *Crataegus chlorocarpa* | Tree | 7 | White | Poem | Insect | 121 ± 4.78 | 137 ± 5.25 | 16 ± 0.47 |
| *Sorbus tianschanica* | Tree | 5 | White | Poem | Insect | 122 ± 5.44 | 133 ± 5.91 | 11 ± 0.94 |
| *Pyrus ussuriensis* | Tree | 15 | White | Poem | Insect | 108 ± 2.05 | 119 ± 3.68 | 11 ± 1.70 |
| *Pyrus betulifolia* | Tree | 10 | White | Poem | Insect | 117 ± 7.79 | 130 ± 4.03 | 13 ± 3.77 |
| *Amygdalus davidiana* | Tree | 10 | Red | Drupe | Insect | 101 ± 6.65 | 113 ± 5.25 | 12 ± 2.16 |
| *Amygdalus triloba* | Tree | 3 | Red | Drupe | Insect | 105 ± 1.00 | 117 ± 7.50 | 12 ± 6.50 |
| *Armeniaca vulgaris* | Tree | 12 | Red | Drupe | Insect | 102 ± 3.09 | 115 ± 2.94 | 13 ± 0.47 |
| *Prunus salicina* | Tree | 12 | White | Drupe | Insect | 108 ± 5.91 | 120 ± 4.11 | 12 ± 2.45 |
| *Padus maackii* | Tree | 10 | White | Drupe | Insect | 112 ± 4.78 | 129 ± 3.09 | 17 ± 2.05 |
| *Cerasus tomentosa* | Shrub | 3 | White | Drupe | Insect | 104 ± 4.00 | 114 ± 3.00 | 10 ± 1.00 |
| *Cerasus tianshanica* | Shrub | 1.5 | Red | Drupe | Insect | 102 ± 2.50 | 113 ± 2.50 | 11 ± 0.00 |
| *Ulmus pumila* | Tree | 25 | Brown | Samara | Wind | 92 ± 0.00 | 100 ± 0.00 | 8 ± 0.00 |
| *Caragana arborescens* | Tree | 6 | Yellow | Pod | Insect | 116 ± 1.25 | 134 ± 3.40 | 18 ± 2.87 |
| *Malus prunifolia* | Tree | 12 | White | Poem | Insect | 110 ± 4.78 | 124 ± 3.74 | 14 ± 1.25 |
| *Euonymus phellomanus* | Shrub | 4 | Green | Capsule | Wind | 135 ± 10.00 | 150 ± 14.50 | 15 ± 4.50 |
| *Acer negundo* | Tree | 20 | Green | Nut | Insect | 99 ± 5.10 | 109 ± 6.38 | 10 ± 2.16 |
| *Acer mono* | Tree | 20 | White | Samara | Insect | 113 ± 2.50 | 128 ± 3.50 | 15 ± 1.00 |
| *Forsythia suspensa* | Shrub | 1 | Yellow | Capsule | Insect | 107 ± 5.25 | 130 ± 2.94 | 23 ± 2.50 |
| *Forsythia ovata* | Shrub | 1.5 | Yellow | Capsule | Insect | 101 ± 1.50 | 119 ± 2.00 | 18 ± 3.50 |
| *Syringa villosa* | Shrub | 4 | Red | Capsule | Insect | 123 ± 2.00 | 141 ± 0.00 | 18 ± 2.00 |
| *Syringa oblata* | Shrub | 5 | Purple | Capsule | Insect | 110 ± 7.72 | 131 ± 4.32 | 21 ± 3.40 |
| *Viburnum opulus* | Shrub | 4 | White | Drupe | Insect | 122 ± 4.32 | 139 ± 1.41 | 17 ± 4.97 |
| *Sambucus williamsii* | Tree | 6 | White | Drupe | Insect | 121 ± 4.78 | 135 ± 1.25 | 14 ± 4.97 |
| *Lonicera tatarica var. micrantha* | Shrub | 3 | White | Berry | Insect | 129 ± 4.00 | 139 ± 3.50 | 10 ± 0.50 |
| *Lonicera maackii* | Shrub | 6 | First white and then yellow | Berry | Insect | 127 ± 2.49 | 142 ± 2.62 | 15 ± 1.25 |
| *Swida alba* | Shrub | 3 | White | Drupe | Insect | 116 ± 1.25 | 132 ± 2.05 | 16 ± 0.82 |
| *Berberis ferdinandi-coburgii* | Shrub | 2 | Yellow | Berry | Insect | 121 ± 6.50 | 143 ± 2.50 | 22 ± 4.00 |
| *Spiraea salicifolia* | Shrub | 2 | Red | Follicle | Insect | 138 ± 0.00 | 156 ± 0.00 | 18 ± 0.00 |
| *Ulmus laciniata* | Tree | 27 | Brown | Samara | Wind | 103 ± 1.00 | 111 ± 1.50 | 8 ± 0.50 |
| *Ulmus pumila cv. pendula* | Tree | 25 | Brown | Samara | Wind | 103 ± 0.00 | 110 ± 0.00 | 7 ± 0.00 |
| *Ulmus densa* | Tree | 20 | Brown | Samara | Wind | 118 ± 4.00 | 130 ± 3.50 | 12 ± 0.50 |
| *Ulmus macrocarpa* | Tree | 20 | Brown | Samara | Wind | 104 ± 0.00 | 110 ± 0.00 | 6 ± 0.00 |
| *Hemiptelea davidii* | Tree | 10 | Brown | Nut | Wind | 117 ± 0.00 | 126 ± 0.00 | 9 ± 0.00 |
| *Rhamnus diamantiaca* | Shrub | 10 | Green | Drupe | Insect | 131 ± 0.00 | 144 ± 0.00 | 13 ± 0.00 |
| *Hippophae rhamnoides* | Shrub | 5 | Orange | Nut | Bird | 110 ± 0.00 | 120 ± 0.00 | 10 ± 0.00 |
| *Elaeagnus angustifolia* | Tree | 10 | White | Nut | Bird | 142 ± 0.00 | 159 ± 0.00 | 17 ± 0.00 |
| *Betula platyphylla* | Tree | 27 | Brown | Nut | Wind | 104 ± 0.00 | 118 ± 0.00 | 14 ± 0.00 |
| *Quercus robur* | Tree | 40 | Yellow | Nut | Wind | 105 ± 0.00 | 116 ± 0.00 | 11 ± 0.00 |
| *Robinia pseudoacacia* | Tree | 25 | White | Pod | Insect | 131 ± 1.50 | 140 ± 1.00 | 9 ± 0.50 |
| *Caragana rosea* | Shrub | 1 | Yellow | Pod | Insect | 117 ± 0.00 | 131 ± 0.00 | 14 ± 0.00 |
| *Halimodendron halodendron* | Shrub | 2 | Red | Pod | Insect | 151 ± 0.00 | 161 ± 0.00 | 10 ± 0.00 |
| *Amorpha fruticosa* | Shrub | 4 | Purple | Pod | Insect | 141 ± 0.00 | 155 ± 0.00 | 14 ± 0.00 |
| *Populus alba var. pyramidalis* | Tree | 30 | Green | Capsule | Wind | 101 ± 0.00 | 111 ± 0.00 | 10 ± 0.00 |
| *Populus nigra var. thevestina* | Tree | 30 | Green | Capsule | Wind | 100 ± 0.50 | 113 ± 1.00 | 13 ± 1.00 |
| *Salix matsudana f. umbraculifera* | Tree | 18 | Green | Capsule | Wind | 112 ± 0.00 | 121 ± 0.00 | 9 ± 0.00 |
| *Salix matsudana* | Tree | 18 | Green | Capsule | Wind | 112 ± 0.00 | 120 ± 0.00 | 8 ± 0.00 |
| *Salix babylonica* | Tree | 18 | Green | Capsule | Wind | 110 ± 0.50 | 121 ± 1.00 | 11 ± 0.50 |
| *Acer ginnala* | Tree | 6 | White | Nut | Insect | 133 ± 0.00 | 145 ± 0.00 | 12 ± 0.00 |
| *Catalpa speciosa* | Tree | 10 | White | Capsule | Insect | 153 ± 0.00 | 171 ± 0.00 | 18 ± 0.00 |
| *Fraxinus chinensis* | Tree | 12 | Yellow | Nut | Insect | 105 ± 1.50 | 117 ± 1.00 | 12 ± 0.50 |
| *Fraxinus mandschurica* | Tree | 30 | Yellow | Samara | Insect | 106 ± 1.00 | 115 ± 0.50 | 9 ± 0.50 |
| *Syringa reticulata var. amurensis* | Shrub | 15 | White | Capsule | Insect | 153 ± 0.00 | 171 ± 0.00 | 18 ± 0.00 |
| *Lycium chinense* | Shrub | 1 | Purple | Berry | Insect | 135 ± 0.00 | 143 ± 0.00 | 8 ± 0.00 |
| *Lonicera tatarica* | Shrub | 3 | Red | Berry | Insect | 135 ± 0.00 | 146 ± 0.00 | 11 ± 0.00 |
| *Tamarix chinensis* | Tree | 8 | Red | Capsule | Insect | 120 ± 0.00 | 136 ± 0.00 | 16 ± 0.00 |
| *Juniperus rigida* | Tree | 10 | Brown | Cone | Wind | 128 ± 0.00 | 137 ± 0.00 | 9 ± 0.00 |
| *Pinus sylvestris* | Tree | 25 | Brown | Cone | Wind | 123 ± 1.00 | 133 ± 1.00 | 10 ± 1.50 |
